# In Situ Synthesis of Silver Nanoparticles in a Hydrogel of Carboxymethyl Cellulose with Phthalated-Cashew Gum as a Promising Antibacterial and Healing Agent

**DOI:** 10.3390/ijms18112399

**Published:** 2017-11-12

**Authors:** Ana Karina Marques Fortes Lustosa, Antônia Carla de Jesus Oliveira, Patrick Veras Quelemes, Alexandra Plácido, Francilene Vieira da Silva, Irisdalva Sousa Oliveira, Miguel Peixoto de Almeida, Adriany das Graças Nascimento Amorim, Cristina Delerue-Matos, Rita de Cássia Meneses de Oliveira, Durcilene Alves da Silva, Peter Eaton, José Roberto de Souza de Almeida Leite

**Affiliations:** 1 Center for Biodiversity Research and Biotechnology, Biotec, Federal University of Piauí, Av. São Sebastião, 2819, Reis Veloso, 64202-020 Parnaíba-PI, Brazil; ana_lustosa@uol.com.br (A.K.M.F.L.); carlinha_nere@hotmail.com (A.C.d.J.O.); pquelemes@gmail.com (P.V.Q.); adriany1210@gmail.com (A.d.G.N.A.); durcileneas@gmail.com (D.A.d.S.); peter.eaton@fc.up.pt (P.E.); 2 REQUIMTE/LAQV, Superior Engineering Institute of Porto, Polytechnic Institute of Porto, Rua Dr. António Bernardino de Almeida, 431, 4200-072 Porto, Portugal; alexandra.placido@gmail.com (A.P.); cmm@isep.ipp.pt (C.D.-M.); 3 Medicinal Plants Reserarch Center, NPPM, Federal University of Piauí, Campus Ministro Petrônio Portella, Bairro Ininga, 64049-550 Teresina- PI, Brazil; francilenev@gmail.com (F.V.d.S.); iryssou@gmail.com (I.S.O.), menesesoliveira@gmail.com (R.d.C.M.d.O.); 4 REQUIMTE/LAQV, Department of Chemistry and Biochemistry, Faculty of Sciences of the University of Porto, Rua do Campo Alegre, 4169-007 Porto, Portugal; mpda@fc.up.pt; 5 Area Morphology, Faculty of Medicine, University of Brasília (UnB), University campus Darcy Ribeiro, Asa Norte, 70910-900 Brasília-DF, Brazil

**Keywords:** silver nanoparticles, hydrogel, cashew gum, healing, wounds, antibacterial activity

## Abstract

Silver nanoparticles have been shown to possess considerable antibacterial activity, but in vivo applications have been limited due to the inherent, but low, toxicity of silver. On the other hand, silver nanoparticles could provide cutaneous protection against infection, due to their ability to liberate silver ions via a slow release mechanism, and their broad-spectrum antimicrobial action. Thus, in this work, we describe the development of a carboxymethyl cellulose-based hydrogel containing silver nanoparticles. The nanoparticles were prepared in the hydrogel in situ, utilizing two variants of cashew gum as a capping agent, and sodium borohydride as the reducing agent. This gum is non-toxic and comes from a renewable natural source. The particles and gel were thoroughly characterized through using rheological measurements, UV-vis spectroscopy, nanoparticles tracking analysis, and transmission electron microscopy analysis (TEM). Antibacterial tests were carried out, confirming antimicrobial action of the silver nanoparticle-loaded gels. Furthermore, rat wound-healing models were used and demonstrated that the gels exhibited improved wound healing when compared to the base hydrogel as a control. Thus, these gels are proposed as excellent candidates for use as wound-healing treatments.

## 1. Introduction

The development of biotechnological devices made with nanoparticles is increasingly promising owing to their application in medical and hospital materials, and their efficiency in preventing and treating wounds, burns, and infections [1]. The antimicrobial action of silver has been known for a very long time [2], and is closely related to the ability of the metal to increase the availability of the oxidised ionic form Ag^+^ in solution. Silver in the form of nanoparticles has been widely investigated as a possible antibacterial agent, although the exact mechanism of action is not yet clear, since it most likely acts in a number of ways, including interactions with thiol and amine groups in proteins, nucleic acids, and cell membranes [3]. Furthermore, some studies have indicated that nanoparticles that can be more efficient than silver ions, possibly related to the possibility of multiple active species, namely action via silver ions, and also by direct contact with the metal [3,4]. However, the use of silver nanoparticles as a replacement for conventional antibiotics is not recommended due to apparent toxicity issues, although there are conflicting reports on this, possibly due to the wide variety of nanoparticles tested [5].

The potential of silver nanoparticles (AgNPs) that are associated with gels, which have traditionally been used in the treatment of wounds, has been investigated [6]. In such an application, silver nanoparticles are likely to represent a useful addition to the hydrogel, as they release silver slowly, and cutaneous toxicity is absent [7]. For example, carboxymethyl cellulose is a polymer that forms an amorphous and three-dimensional (3D) hydrogel that is better suited to produce nanoparticles than most aqueous systems. Nanoparticles can either be incorporated into the hydrogel matrix by being mixed with the preformed gel or reduced in situ during the gelation process [6,8]. These hydrogels can serve as a reservoir for the sustained release of silver ions and maintain a moist environment to aid in wound healing. Antimicrobial activity of some such gels has been proven, with wound healing mentioned as a possible application [6,8].

Based on the potential of associating metals with polymers, cashew gum (CG), which is a heteropolysaccharide abundant in northeastern Brazil, has been applied in the synthesis of silver nanoparticles with antimicrobial properties [9,10]. Some modifications of the chemical structure of this carbohydrate have been carried out in order to change different characteristics and increases in reactivity in reactions such as carboxymethylation [11], oxidation [12], sulfation [13], acetylation [14], and quaternization [15]. The application of natural polymers has been important for the stabilization of the colloidal system, as well as for improved biocompatibility [16]. Due to the antimicrobial properties of cashew gum alone [17], as well as its ability to reduce silver to form nanoparticles that are coupled with high biocompatibility [9,10], cashew gum-capped silver nanoparticles appear to be a possibly useful antimicrobial agent with which to load gels for topical applications to wounds. No previous work has demonstrated the production and characterization of hydrogels containing such particles however. Although in vitro antimicrobial activity of cashew-gum capped nanoparticles has been shown, no in vivo work have so far been demonstrated, nor their activity in wound-healing applications.

In this work, we report the synthesis and characterization of AgNPs reduced in situ in hydrogels consisting of carboxymethyl cellulose mixed with natural CG or gum modified with phthalic anhydride. We report on the antimicrobial effects of the resulting silver nanoparticle-loaded gel on two model organisms, and assess the healing properties in rat models.

## 2. Results

**Figure 1:**
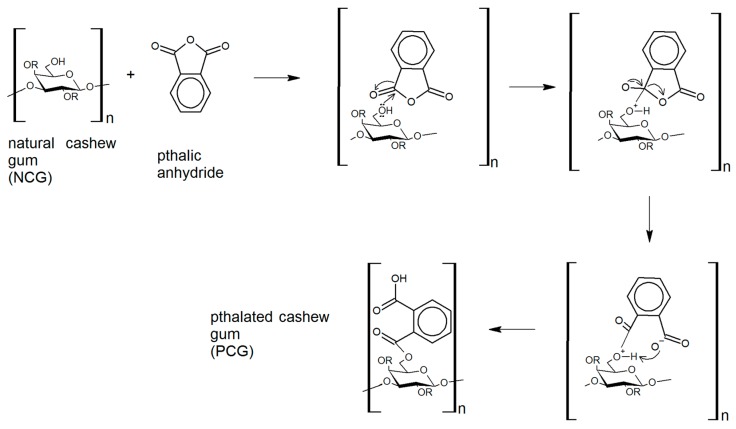
General reaction scheme for pthalation of cashew gum. The reaction proceeds via homogeneous esterification, with no side products.

**Figure 2:**
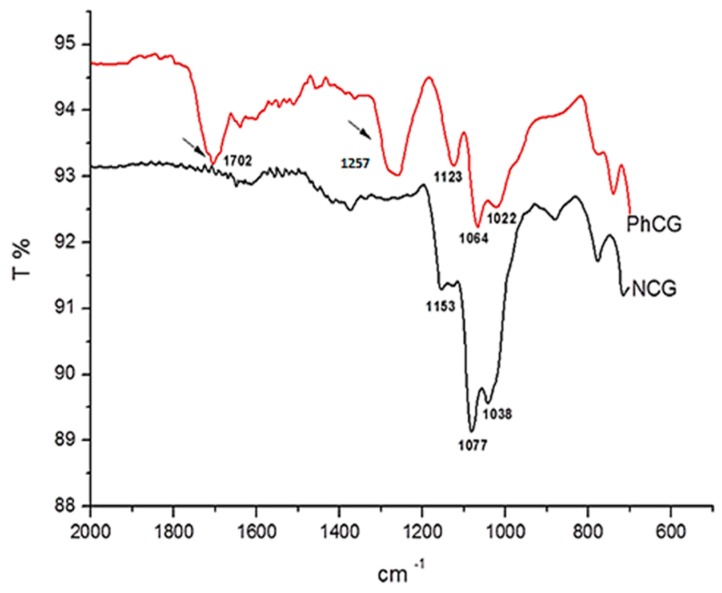
Attenuated total reflection (ATR)—Fourier transform infrared (FTIR) spectra of natural cashew gum (NCG) and phthalated-cashew gum (PhCG). Arrows indicated the new bands due to pthalation (see text).

The pthalation reaction adds several functional groups to the carbohydrate structure that can be probed using Fourier transform infrared spectroscopy (FTIR) (Figure 1). The cashew gum modification reaction success was confirmed by FTIR spectrum analysis, as shown in Figure 2. Infrared spectra showed characteristic bands at 1153, 1078, and 1038 cm^−1^ for natural gum and at 1123, 1064, and 1022 cm^−1^ for phthalated gum, which are attributed to the stretching vibrations of the C–O–C glycoside bonds. The difference that indicates the insertion of the group corresponds to the bands at 1257 and 1702 cm^−1^. The band at 1257 cm^−1^ is attributed to C–O stretching, which is characteristic of an aromatic ester, and the 1702 cm^−1^ band to the C=O stretch. Together, the appearance of these two bands shows the successful incorporation of the new group into the polysaccharide structure, as illustrated in Figure 1.

**Figure 3:**
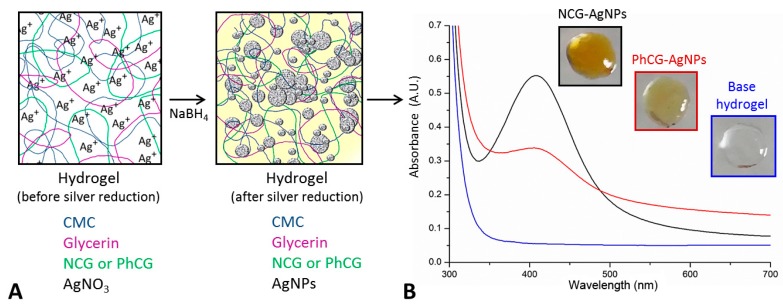
(**A**) Scheme of the in situ synthesis of silver nanoparticles (AgNPs), using cashew gum (CG) and PhCG; (**B**) UV-Vis spectra and photographs of NCG-AgNPs and PhCG-AgNPs.

In situ AgNPs formation was observed by color change, from clear to yellow after the addition of the NaBH_4_ (as reducing agent), to previously prepared hydrogels containing AgNO_3_ (Figure 3A). In the gel containing natural cashew gum (NCG), a more intense color was observed, when compared to that seen in the case of PhCG-AgNPs, as shown in Figure 3B.

The formation of the nanoparticles manufactured in situ in CMC hydrogels was confirmed by UV-Vis spectroscopy analysis, with the formation of plasmonic bands in the region between 380 and 420 nm (Figure 3B) that are characteristic of AgNPs [18]. For the NCG-AgNPs hydrogel, the observed plasmon band was centered at 408 nm, while for the PhCG-AgNPs gel, the maximum absorbance was at 404 nm. A higher intensity in the plasmon band, together with lower absorbance in the 500–700 nm region, was observed for the NCG-AgNPs, indicating the formation of a more monodisperse sample. 

**Figure 4:**
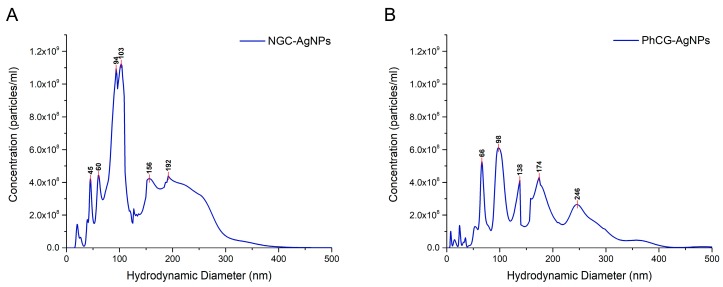
Quantitative particle size analysis by nanoparticle tracking analysis (NTA). Merged results for samples (**A**) NCG-AgNPs (Mean: 119.7 ± 5.1 nm and Mode: 86.4 ± 4.7 nm, 6.36 × 10^10^ particles·mL^−1^) and (**B**) PhCG-AgNPs (Mean: 123.8 ± 8.9 nm and Mode: 103.1 ± 18.8 nm, 4.03 × 10^10^ particles·mL^−1^).

The nanoparticle tracking analysis (NTA) results suggest that the mean size of the NCG-AgNPs was 119.7 ± 5 nm and the concentration was 6.36 × 10^10^ particles·mL^−1^, while for PhCG-AgNPs, the mean size was 123.8 ± 8.9 nm with a concentration of 4.03 × 10^10^ particles·mL^−1^ (Figure 4). The very small difference between the mean particle sizes corroborates the UV-vis data discussed above, indicating average sizes to be similar. The small difference in the number of particles corroborates the results of the UV-Vis analysis where the plasmon band was more intense for the natural cashew gum (NCG) sample. However, observation of Figure 4 shows that a broad dispersion of sizes was found for both samples, ranging from 20 to 250 nm for the NCG-AgNPs, and from 7 to 500 nm for the PhCG-AgNPs. Since this sample was a concentrated gel, considerable dilution was necessary to perform NTA analysis (a procedure that is necessary for the vast majority of nanoparticle samples), which it appears lead to a variety of species in solution. These can be due to nanoparticle aggregates, as well as aggregated and free polymer in solution.

The zeta potential analysis showed that hydrogel base, NCG-AgNPs, and PhCG-AgNPs presented the following values: −70.7 (standard deviation, SD = 3.47); −78.9 (SD = 3.47) and −77.5 (SD = 3.06), respectively. Overall, these differences are not very significant, but all of the samples had extremely high negative zeta-potentials, which should indicate good colloidal stability.

**Figure 5:**
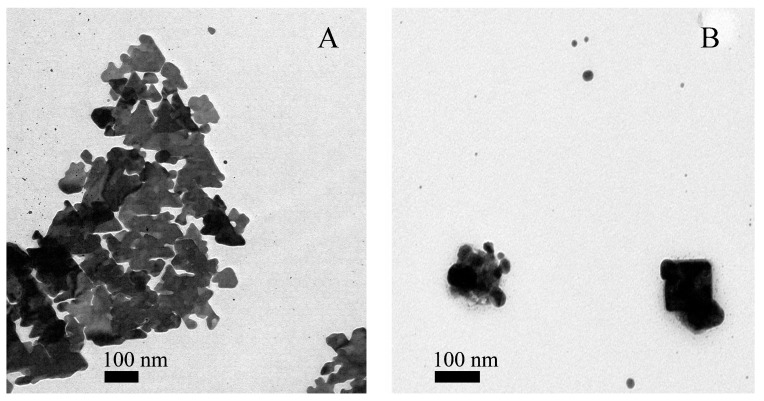
Representative transmission electron microscopy (TEM) images of AgNPs. (**A**) NCG-AgNPs; (**B**) PhCG-AgNPs.

In this work, transmission electron microscopy (TEM) was performed to determine the morphology of the AgNPs formed in the hydrogels. Figure 5A shows the formation of triangular AgNPs, double triangles, and hexagons, for the nanoparticles formed with NCG; and, Figure 5B shows a somewhat more heterogeneous sample with the presence of spherical, square, and aggregates of irregular shapes formed with PhCG. It has been noted previously that the shape of the nanoparticles can be directly determined by the stabilizing agent, as in the case of NCG and PhCG [10]. The energy dispersive spectroscopy (EDS) spectra that were obtained in the TEM (Tables S1 and S2) showed strong silver signals, in both cases. The presence of silver-bound peaks confirms the presence of nanoparticles in the NCG-AgNPs and PhCG-AgNPs hydrogels. It is worth noting that these relatively high-contrast features were observed with a light grey “halo” in the low-magnification TEM images, which presumably corresponded to the hydrogel.

**Figure 6:**
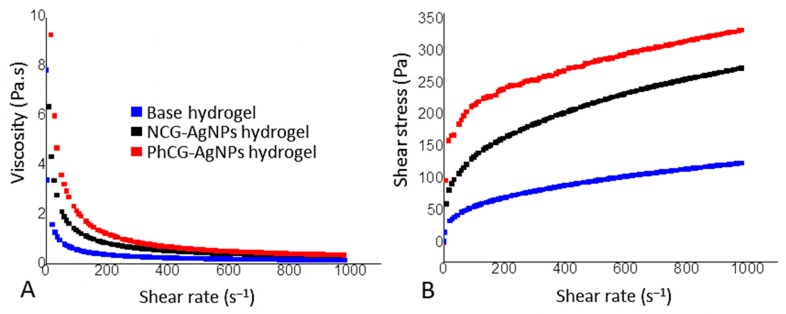
Rheological analysis of hydrogels formulated with AgNPs: Viscosity (**A**) and Shear stress (**B**).

Rheological analysis was performed to observe the viscoelastic parameters of the samples. Carboxymethyl cellulose gels are considered non-Newtonian and pseudoplastic, since there is a decrease in viscosity as the shear rate increases. Indeed, this was observed for both hydrogel samples, those containing NCG-AgNPs and PhCG-AgNPs (Figure 6A). It was observed that after handling, the gels prepared with CMC and NCG or PhCG with the nanoparticles, the mechanical properties underwent changes. Hydrogels with PhCG-AgNPs and NCG-AgNPs were more consistent with respect to the base hydrogel. This can be explained by the fact that the gums, even in small concentrations, promote an increase in the viscosity of the systems. The PhCG-AgNPs hydrogel showed a higher shear stress (Figure 6B) suggesting an interaction between the chemical structures of the CMC and the PhCG, since the two are negatively charged and may exhibit more repulsion.

For this study, to verify the antibacterial activity of hydrogels formulated with AgNPs, their effects on two bacterial species, *S. aureus* and *P. aeruginosa*, in infectious wound processes were examined.

**Figure 7:**
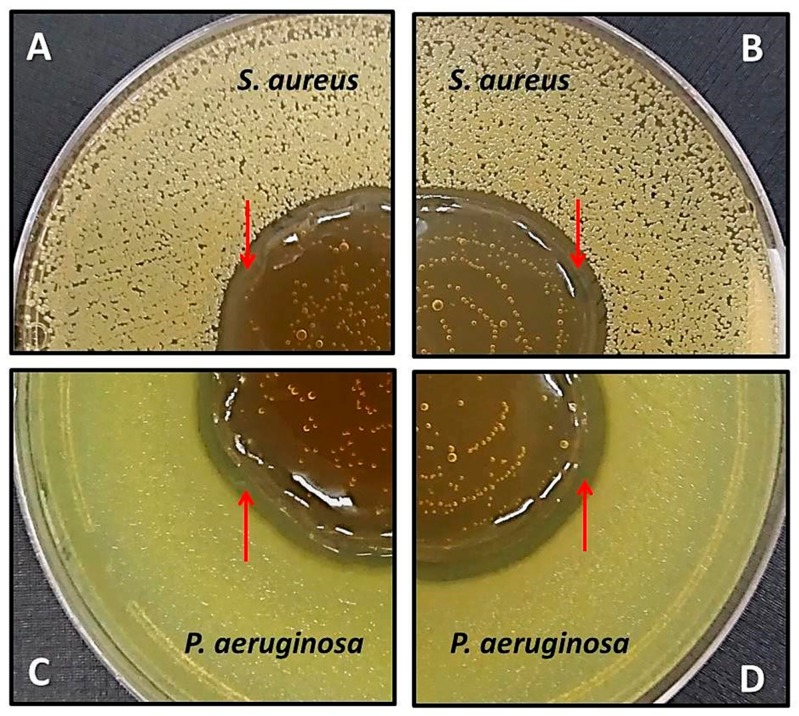
Direct contact antibacterial effect of NCG-AgNPs (**A**,**C**) and PhCG-AgNPs (**B**,**D**) hydrogels, with the presence of halos of inhibition indicated by arrows.

Initially, the effect of direct contact was evaluated when no bacterial growth was observed under the hydrogels after 24 h incubation. Formation of halos of inhibition around the deposited hydrogels for both microorganisms was also observed (Figure 7). The hydrogel base did not cause any inhibitory effect.

Further studies of MIC and MBC determination showed a potent effect of hydrogels with AgNPs on the bacteria tested (Table 1). The hydrogel base did not present an antimicrobial effect, and the standard antibiotics used as controls showed the effects predicted by the CLSI. In general, the effect of the hydrogels was more effective against *P. aeruginosa*, whereas the hydrogel PhCG-AgNPs was more potent than the NCG-AgNPs. For the gram-negative bacterium, the MIC values presented the same value of MBC for both hydrogels, which indicates a bactericidal effect. For example, MIC and MBC of PhCG-AgNPs on *P. aeruginosa* were both 0.84 μg Ag·mL^−1^. According to Venkatpurwar and Porkharkar [19], this effect of AgNPs on gram-negative bacteria can be explained by the rapid internalization of the nanoparticles through the cell walls of these bacteria, which contain low concentrations of peptidoglycan, and inactivating and/or altering protein structures, thus leading to cell death.

Hydrogels with AgNPs showed lower MICs when compared to the effect of AgNO_3_ solutions at the same concentrations tested for the two bacteria. For example, the effect of PhCG-AgNPs on *S. aureus* caused a MIC of 3.37 μg Ag·mL^−1^, whereas AgNO_3_ needed a 4-fold higher concentration of silver to inhibit bacterial growth (Table 1). 

**Figure 8:**
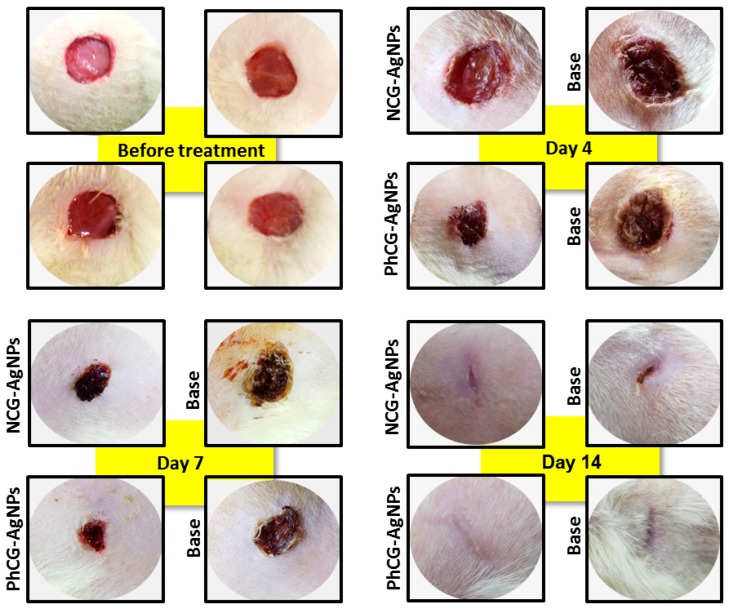
Representative images of the healing process caused by AgNPs-hydrogels on the back of rats models.

**Figure 9:**
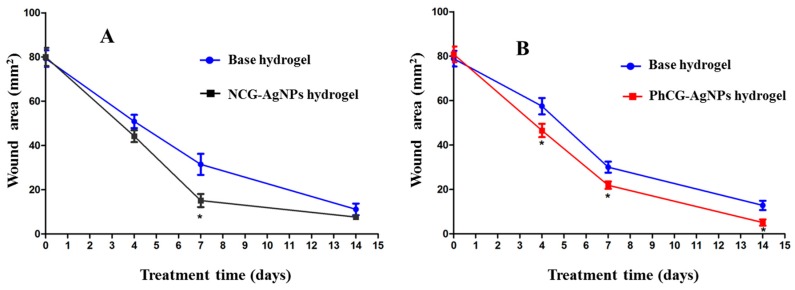
Relation between the time of action of the NCG-AgNPs (**A**) and PhCG-AgNPs (**B**) hydrogels and the wound area in rats. * *p* < 0.05 statistical significance.

In addition to the potent antimicrobial activity, products containing AgNPs have recognized the auxiliary ability in the wound healing process [20]. In this work, direct observation of the wounds (Figure 8) was accompanied by statistical analysis of the lesion sizes (Figure 9). This analysis showed that over the first of 4 postoperative days, there was no significant difference between the means of contraction rates of the wounds of the NCG-AgNPs group (47 ± 3.2 mm^2^) when compared to the base formulation group (52 ± 2.8 mm^2^). However, the mean wound contraction index was significantly higher in the group treated with the formulations containing PhCG-AgNPs (43 ± 3.0 mm^2^, *p* < 0.05) when compared to that in the group with the base formulation (62 ± 3.9 mm^2^).

At seven days after surgery, a significant difference was observed between the mean contraction rates of the wounds of the group treated with the formulations containing NCG-AgNPs (16 ± 3.1 mm^2^, *p* < 0.05), as well as the formulation containing PhCG-AgNPs (20 ± 1.9 mm^2^, *p* < 0.05) when compared to that in groups that received only the base formulation (31 ± 4.7 mm^2^, 33 ± 3.0 mm^2^).

At 14 days after surgery, there was no significant difference between the mean contraction rates of the wounds of the animals containing the formulation with NCG-AgNPs (7 ± 0.5 mm^2^) when compared to the base formulation group (11 ± 1.6 mm^2^). However, the mean rate of wound contraction was significantly higher in the group treated with PhCG-AgNPs (5 ± 0.8 mm^2^, * *p* < 0.05) when compared to that in the base formulation group (12 ± 1.4 mm^2^).

## 3. Discussion

CG was chosen as the capping agent for the AgNPs because this polymer has been previously applied in AgNP synthesis, and showed antimicrobial activity [9,10]. In addition, cashew gum itself is highly non-toxic and shows mild antibacterial activity [10,17]. Several types of chemical modifications have previously been used to treat CG in order to improve its technological properties [21]. In this work, NCG was modified by a reaction using the solvent phthalic anhydride. The modification occurred by means of a nucleophilic addition reaction to the hydroxyl group of the carbon 6 of the galactose unit, leading to the formation of an ester/acid group, and consequently the presence of a remaining carboxyl group.

In the case of the PhCG-AgNPs, the lower ratio between the intensities of the plasmonic band and the whole 500–700 region point to a higher percentage of larger nanoparticles (or particle aggregates) in the sample and/or to a multiplicity of shapes. It is likely that aggregates are present, since both samples seem to have individual nanoparticles that are close in size, based on the wavelength of the plasmonic band maxima, which only differ by a few nanometers. The in situ nanoparticle formation reaction was attributed to the reduction of Ag^+^ silver to Ag0, by the NaBH_4_ reducing agent and also possibly by the gums used, which can also have a weak reducing potential [22].

Depending on the technique used to synthesize AgNPs, it is possible to form diverse particles of different sizes and shapes. Spherical, hexagonal, triangular, and bar-shaped nanostructures have been previously synthesized using CMC, all by reducing Ag^+^ at different temperatures [23].

Despite the gums being present in small concentrations, they did significantly alter the rheological properties of the hydrogels. Hydrogels with PhCG-AgNPs and NCG-AgNPs were more viscous, and stiffer at all of the shear rates tested, than the base hydrogel. The PhCG-AgNPs hydrogel showed a higher shear stress (Figure 6B), suggesting a stronger interaction between the CMC and the PhCG, compared to the natural gum.

When considering that the arsenal of drugs is limited by pathogenic bacteria′s ability to resist antibacterial agents, the development of new products using nano-sized silver particles as an antimicrobial agent is an important technological goal [24]. In this context, the antimicrobial effect of AgNPs in CMC gels has been previously reported [6].

Antimicrobial properties of CG-capped AgNPs have also been previously studied in our group. Using the same technique to determine the MIC and comparing our results with those presented by Quelemes et al. [10], it was observed that the antimicrobial effect of the AgNPs synthesized in the hydrogel was more potent than the effect of the cashew gum-coated AgNPs in aqueous solution that was developed in that study. For example, the CIM and CBM of the NCG-AgNPs hydrogel against *P. aeruginosa*, as reported here, presented values equal to 1.68 μg Ag·mL^−1^ and the values of the effect of the AgNPs in solution from Quelemes et al., stabilized with CG were 3.37 μg Ag·mL^−1^ and 6.65 μg Ag·mL^−1^, respectively [10]. The same trend occurred for *S. aureus*. This increase in antimicrobial capacity can be explained by a more effective ionic silver reduction process, as proposed in this work, in which a strong reducing agent, sodium borohydride, was used, or alternatively by a better stabilizing effect that was caused by the constituents of the gel; namely CMC and glycerin. It is worth noting that in previous work [10], cytotoxicity of very similar nanoparticles to those that were used in this work was measured, and was observed at concentrations slightly higher than the inhibitory concentrations of the nanoparticles. The cytotoxicity was also slightly lower than that observed for the starting silver compound. Although these results are promising, worries about toxicity are likely to limit use of silver nanoparticles via oral while we might expect wound healing applications to be more likely to be tolerated.

Wounds, particularly non-healing wounds, are a major health problem worldwide, and can lead to remarkable morbidity, prolonged treatment time, and high health-care costs. Skin lesions can arise from several inflammatory processes that occur in the skin due to burns or inflammatory diseases [25,26]. Wound healing involves an orderly progression of restoring the integrity of damaged tissue, including inflammation, proliferation, and remodeling [27]. The inflammation phase begins immediately after injury, with an initial vasoconstriction that favors homeostasis and the release of inflammatory mediators. The proliferative phase is characterized by proliferation of granulated tissue that is formed mainly by fibroblasts and by the angiogenesis process. Remodeling involves a series of complex regenerative reactions, triggered by biochemical signaling and inflammatory mediators, originating from the rupture of tissue continuity.

In the present study, it was possible to identify an acceleration of wound healing by treatment with these gels (Figure 8), which has potential to contribute to the search for therapeutic alternatives for wound healing. Most studies of cutaneous pathologies for the treatment and healing of wounds have used mouse models that present histological characteristics that resemble the human skin. Inflammatory and cutaneous processes are most often reproduced in mice and rats.

Our study was based on macroscopic evaluation, and although this method is very subjective, it is of great importance for the follow-up of the repair of surgical wounds and generates important information regarding the biological events during tissue repair [28].

In the analysis of wound contraction, there was a progressive decrease in the lesion area in all of the groups; no wound presented a greater area than the initial one, and the area decreased owing to the mechanism of tissue contraction. It was also observed that the percentages of wound contractions were higher after seven days; this was expected, since the fibroplasia phase of healing occurs between seven and 14 days, causing the presence of fibroblasts and myofibroblasts.

After 14 days of treatment, it was observed that in the group containing the PhCG-AgNPs formulation, the healing was superior when compared to that with the formulation containing NCG-AgNPs. The gel containing PhCG-AgNPs was effective at all of the stages of healing evaluation, while NCG AgNPs showed significant differences after only four days of treatment, suggesting that the PhCG-AgNPs gel was able to accelerate the healing process, possibly because of the effect of CG on various factors that act in the inflammatory process, cell migration, and tissue repair.

In the groups that were treated with both formulations containing CG after 14 days of treatment, the exudate was absent or present in very small quantities. The exudate, when present, presented a serous appearance, which is characteristic of uncontaminated wounds. In the groups with base formulations, the presence of exudate persisted up to day nine. In acute wounds, the presence of exudate is normal during the first 48 and 72 h, however, after this time, the presence of exudate is considered detrimental to healing [29]. The daily treatment with NCG-AgNPs and PhCG-AgNPs showed progressive evolution of scarring and gradual reduction of exudate.

Hyperemia is characterized as a vascular phenomenon involved in the inflammation process during tissue repair, beginning a few min after the traumatic event, at which time several chemical mediators, such as histamine, are released [27]. In this work, hyperemia was verified in all of the groups, varying only in their intensity. In the groups treated with NCG-AgNPs and with PhCG-AgNPs showed intense hyperemia, especially in the first three days. In the group treated with NCG-AgNPs, the hyperemia lasted longer, persisting up to the ninth day, while in the group treated with PhCG-AgNPs, after the third day hyperemia became mild and then discrete or absent on the remaining days of treatment. This demonstrated that there was significant action in the control of upper inflammatory reaction to PhCG-AgNPs when compared to NCG-AgNPs. In the groups treated with the base formulations, this phenomenon persisted up to 10 days.

## 4. Materials and Methods

### 4.1. CG Modification 

CG was isolated from the exudate from trees of the genus *Anacardium occidentale L*., native to Parnaíba, Piauí, Brazil. CG was purified by precipitation in ethyl alcohol by the method described by Dias et al. [30]. The gum modification was carried out following the methodology proposed by Vieira et al. [31], with some modifications. This is carried out by a direct homogeneous esterification in the absence of solvent. Briefly, 1 g CG and 5 g phthalic anhydride (PA) were used. The PA was melted in an oil bath at 131 °C with constant stirring, and then CG was added and the reaction was carried out over for 40 min at 131 °C with stirring. After this time, *N*,*N*-dimethylacetamide (5 mL) was added to stop the reaction. The product of the reaction was precipitated and washed with ultrapure water to remove by-products.

### 4.2. Preparation In Situ of AgNPs in Hydrogel 

Nanoparticles were prepared in situ during the manufacture of the hydrogel. Firstly, a hydrogel containing neither nanoparticles nor cashew gum was prepared to act as a control, and is referred to henceforth as hydrogel base. This was prepared using 2.0 g of carboxymethylcellulose (CMC) and 5.0 g of glycerin that made up to a total weight of 100 g with ultrapure water. It was prepared using an ultraturax homogenizer for 10 min. Gels containing nanoparticles likewise used 2.0 g CMC, and 5.0 g glycerin, with the addition of 0.2 g of natural cashew gum (NCG) or 0.2 g phthalated-cashew gum (PhCG). In addition these mixtures contained 0.0170 g silver nitrate (1 × 10^−4^ mol), and were also made up to 100 g total weight using ultrapure water. This base was homgenised for 5 min, before the addition of 0.0227 g of sodium borohydride (6 × 10^−4^ mol), to give a molar ratio of 6:1 in reductant to metal complex. The hydrogel was then further homogenized for a further 5 min. Hydrogels containing AgNPs with natural CG (NCG-AgNPs) and a hydrogel containing AgNPs with modified CG (PhCG-AgNPs) were produced.

### 4.3. FTIR-ATR Spectroscopy 

The identification of the functional groups of NCG and PhCG were investigated by Fourier transform infrared (FTIR) using a Thermo Nicolet 6700 spectrometer, with the attenuated total reflection (ATR) technique in the spectral range from 4000 to 700 cm^−1^. The powdered samples were placed onto the ATR crystal and the sample spectrum was collected. 

### 4.4. UV-Vis Spectral Analysis

The hydrogel base, and the NCG-AgNPs and PhCG AgNPs hydrogels were each diluted 1:1 in ultrapure water, and their UV-Vis spectra were analyzed using a spectrophotometer (UV-3101 PC, Shimadzu, Japan). The solutions formed were analyzed between the wavelengths 300 of 700 nm.

### 4.5. Nanoparticle Tracking Analysis 

Nanoparticle tracking analysis (NTA) was carried out using a using a NanoSight NS300 instrument, with a 642 nm laser module and NTA 3.2 software (Malvern Instruments, Malvern, UK), to obtain the diameter and the concentration of particles that were suspended in the feed. An aliquot was taken using a plastic syringe and was injected slowly into the sample chamber (~1 mL). Ensuring that there were no visible air bubbles and no particles adhered to the chamber walls, the focus and camera level were adjusted to obtain the best possible view of the particles, following the guidelines that were provided by the manufacturer. Five videos each of 1-min length were captured; the sample was advanced to ensure that a previously unmeasured set of nanoparticles would be captured by the camera before starting each video. This allowed the measurement of a larger number of different particles across the aliquot. The analysis settings, in particular the detection threshold, were set depending on the scattered light intensity that was observed in the captured videos. The viscosity values of the medium were adjusted independently, according to experimental data from the formulation. For all of the formulations, 1:100 dilutions were made in ultrapure water. Each video was analyzed independently, and the results were automatically merged into one particle size distribution chart.

### 4.6. Zeta Potential Measurements

Zeta potential (ζ) was measured at 25 °C by dynamic light scattering (DLS). Aliquots of NCG-AgNPs and PhCG-AgNPs hydrogel semi-solid formulations were diluted 1:10 in ultrapure water, and analyzed using the Zetasizer Nano ZS (Malvern Instruments Ltd., Malvern, UK). 

### 4.7. Transmission Electron Microscopy and Energy Dispersive Spectroscopy 

For transmission electron microscopy analysis (TEM), 10 µL of samples were deposited on Formvar/carbon film-coated mesh nickel grids (Electron Microscopy Sciences, Hatfield, PA, USA) and were left to stand for 2 min. Excess liquid was removed with filter paper. Visualization was carried out on a JEOL JEM 1400 TEM at 120 kV (Tokyo, Japan). Images were digitally recorded using an Orious 1100 W (Tokyo, Japan) CCD digital camera at the HEMS/i3S of the University of Porto, Porto, Portugal. The energy dispersive spectroscopy (EDS) analysis was performed using a JEOL JED-2300 analysis station with an accelerating voltage of 20 KeV.

### 4.8. Rheology of Gels

Rheological analysis was performed in an AR50 model rheometer from TA Instruments (New Castle, DE, USA). Flow analyses were performed, with a cone-type geometry and a 20 mm diameter plate. The shear rate ranged from 0 to 1000 s^−1^ and the temperature was maintained at 25 °C.

### 4.9. Bacterial Growth and Culture Conditions

To study the antimicrobial effect of hydrogels with AgNPs, *Staphylococcus aureus* ATCC 29123 (gram-positive) and *Pseudomonas aeruginosa* ATCC 27853 (gram-negative) bacteria were reactivated in Mueller-Hinton broth (HIMEDIA), and were incubated at 37 °C for 24 h. From the broth, bacteria were cultured in Mueller-Hinton agar (HIMEDIA) under the same conditions. Then, isolated colonies were collected to prepare a suspension in sterile saline solution (NaCl, 0.9% *w*/*v*) with a turbidity equivalent to a 0.5 McFarland standard (1−2 × 10^8^ CFU/mL). This suspension was further diluted to obtain the desired concentration for the experiments described below.

### 4.10. Antibacterial Contact Assessment

From the diluted solution previously described, bacteria were inoculated on Mueller-Hinton agar (1 × 10^6^ CFU/mL), using sterile swabs, as recommended by the Clinical Laboratory Standards Institute (CLSI, 2013). Soon after, 1 g of each hydrogel with AgNPs at a concentration of 1000 μM or 108 μg Ag·mL^−1^ was deposited on the central area of the inoculated agar and the plates are incubated at 37 °C for 24 h to verify bacterial inhibition resulting from its contact. The hydrogel base was used as a negative control.

### 4.11. Determination of the Minimum Inhibitory Concentration (MIC) and Minimum Bactericidal Concentration (MBC) of Hydrogels with AgNPs

The MIC was determined using a 96-well microdilution plate with Mueller-Hinton broth, in which the strains (5 × 10^5^ CFU/mL) were exposed to 2-fold dilution series of each hydrogel with AgNPs. Silver concentrations ranged from 3.9 to 250 μM (or 0.42 to 27 μg Ag·mL^−1^). As a control, the MIC of AgNO_3_ at the same concentrations previously described was determined, as well as those of standard antibiotics that were effective against these strains. The plates were incubated for 24 h at 37 °C in aerobic conditions. The MIC was defined as the lowest concentration of agent that restricted the visual bacterial growth in the culture media. For the MBC determination, aliquots (10 μL) from all of the wells with concentrations ≥ the MIC concentrations were sub-cultured on Mueller-Hinton agar. The MBC was defined as the lowest concentration that resulted in no growth on the agar. All of the assays were performed in triplicate.

### 4.12. Healing Activity 

In vivo tests were performed on male Wistar rats (180–220 g) that were kept in standard cages at a controlled temperature (24 ± 1 °C) with a light/dark cycle of 12 h, with free access to water and feed. Rats were fasted (solids) for 18 h, and acclimatized to the test environment 2 h before each experiment. The animals were randomly divided into different groups. The animals were anesthetized with a combination of ketamine hydrochloride and xylazine hydrochloride (50 and 5 mg·kg^−1^, intramuscular injection, respectively). After the experimental procedures, the animals were euthanized by sodium thiopental (100 mg·kg^−1^, intraperitoneal injection). All of the experimental protocols were approved by the Animal Ethics Committee of the Federal University of Piauí, Teresina, Brazil (CEEA/UFPI, number 135/16).

Two groups of rats (*n* = 5 per group) were divided into control and experimental groups treated with one dose per wound. In the same animal, two 8 mm incisions (surgical wounds) were performed. One wound (left side) received the hydrogel base, and the other (right side) received the test formulation (NCG-AgNPs or PhCG-AgNPs). The procedures were performed in an aseptic environment, with all autoclaved surgical materials. First, a manual trichotomy was performed in the middle region of the dorsum. For wound induction, a circular metal punch 8 mm in diameter was used in the cervical dorsum region of each animal.

The treatments were applied immediately after surgery and daily thereafter at the same time. Both the base gel and the formulations were applied to the wounds of the animals on days 0, 4, 7, and 14 days after surgery, and the healing process was evaluated. All of the animals were examined daily for macroscopic evaluation of the wound, observing the presence or absence of hemorrhage, exudate, and crust, and the data were recorded in individual files. The wounds were photographed at days 0, 4, 7, and 14 of the experimental protocol, and the area of the wound area was measured. At the end of the chronic treatments, the animals were euthanized with anesthetic overdose (thiopental sodium, 100 mg·kg^−1^).

### 4.13. Statistical Analysis

According to the methodology used in each test, one-way ANOVA followed by the Tukey test for multiple comparisons was used, establishing a significance level of 5% (*p* < 0.05). 

## 5. Conclusions

Chemical modification of CG with phthalic anhydride was confirmed by FTIR spectroscopy. The formation of the AgNPs in the hydrogels was also confirmed. The in vitro antimicrobial activity of the nanoparticle-loaded hydrogels was found to be effective, as observed by the inhibition of bacterial growth and formation of inhibition halos. This was true for both our *S. aureus* and *P. aeruginosa* strains. The antibacterial activity was also confirmed by reduction in MIC and MBC values when compared to silver salt. The in vivo healing activity of the hydrogels was verified by the wound healing model of rats with surgical wounds. It was observed that the CG modified by phthalic anhydride showed the most improved healing profile, as compared to the hydrogel base formulation. Thus, this material represents a promising candidate for wound healing applications.

## Figures and Tables

**Table 1:** Minimum inhibitory concentrations (MIC) and minimum bactericidal concentrations (MBC) of NCG-AgNPs and PhCG-AgNPs hydrogels.

Bacterial Strain	NCG-AgNPs μM (μg Ag·mL^−1^)			PhCG-AgNPs μM (μg Ag·mL^−1^)		AgNO_3_ μM (μg Ag·mL^−1^)	Antibiotic (μg·mL^−1^)
	MIC	MBC		MIC	MBC	MIC	MIC
*S. aureus* ATCC 29213	62.5 (6.75)		250 (27)	31.25 (3.37)	250 (27)	125 (13.5)	Oxacillin < 0.5
*P. aeruginosa* ATCC 27853	15.6 (1.68)		15.6 (1.68)	7.81 (0.84)	7.81 (0.84)	31.25 (3.37)	Meropenem < 0.5

## Supplementary Materials

Supplementary materials can be found at www.mdpi.com/1422-0067/18/11/2399/s1.

## Abbreviations

CG	Cashew Gum
AgNPs	Silver nanoparticles
NCG-AgNPs PhCG-AgNPs	Natural cashew gum-coated silver nanoparticles Pthalated cashew gum-coated silver nanoparticles
PhCG	Pthalated cashew gum
