# Supplementary material for: In Situ Synthesis of Silver Nanoparticles in a Hydrogel of Carboxymethyl Cellulose with Phthalated-Cashew Gum as a Promising Antibacterial and Healing Agent
Source: Int J Mol Sci. 2017 Nov 12;18(11):2399. doi: 10.3390/ijms18112399 (PMC5713367; doi:10.3390/ijms18112399)
Supplement: Supplementary file 1 [file ijms-18-02399-s001.pdf]

Supplementary Information

***In situ* synthesis of silver nanoparticles in a hydrogel of carboxymethyl cellulose with phthalated-cashew gum as a promising antibacterial and healing agent**

Ana Karina Marques Fortes Lustosa[1], Antônia Carla de Jesus Oliveira[1], Patrick Veras Quelemes[1], Alexandra Plácido[2], Francilene Vieira da Silva[3], Irisdalva Sousa Olieviera[3], Miguel Peixoto Almeida[4], Adriany das Graças Nascimento Amorim[1], Cristina Delerue-Matos[2], Peter Eaton[4], Rita de Cássia Meneses de Oliveira[3], Durcilene Alves da Silva[1], José Roberto de Souza de Almeida Leite[5]*

[1]*Center for Biodiversity Research and Biotechnology, Biotec, Federal University of Piauí, Av. São Sebastião, 2819, Reis Veloso, 64202-020, Parnaíba-PI, Brazil*

[2]*REQUIMTE/LAQV, Superior Engineering Institute of Porto, Polytechnic Institute of Porto, Rua Dr. António Bernardino de Almeida, 431, 4200-072 Porto, Portugal*

[3]*Medicinal Plants Reserarch Center, NPPM, Federal University of Piauí, Campus Ministro Petrônio Portella, Bairro Ininga, 64049-550, Teresina- PI, Brazil*

[4]*REQUIMTE/LAQV, Department of Chemistry and Biochemistry, Faculty of Sciences of the University of Porto, Rua do Campo Alegre, 4169-007 Porto, Portugal*

[5]*Area Morphology, Faculty of Medicine, University of Brasília (UnB), University campus Darcy Ribeiro, Asa Norte, 70910-900, Brasília-DF, Brazil*

1. EDS Results.

EDS Spectra were obtained with the electron beam focussed directly on agglomeratoes of nanoparticles within each AgNP-containing hydrogel. The results below indicate a high weight % of silver in the along with a majority of organic material, and some small contamination with iron and silicon.

Table 1: EDS analysis results from sample of hydrogel containing NCG-AgNPs.

Element	Peak Area	Weight %	Atomic %
**C**	29466	9.01	44.64
**O**	836	0.19	0.72
**Si**	20527	2.59	5.49
**Fe**	892	0.14	0.15
**Ag**	388357	87.76	48.43

Table 2: EDS analysis results from sample of hydrogel containing PhCG-AgNPs.

Element	Peak Area	Weight %	Atomic %
**C**	61831	54.04	81.81
**O**	8791	5.79	6.58
**Si**	1190	0.43	0.28
**Fe**	337	0.15	0.05
**Ag**	46605	30.12	5.08
